# Impact of Serum Triglyceride Levels on Severity and Outcomes in Acute Biliary Pancreatitis: A Retrospective Cohort Study

**DOI:** 10.7759/cureus.65928

**Published:** 2024-08-01

**Authors:** Ihtisham ul Haq, Muhammad Daud, Muhammad Attaullah Khan, Fahim Ullah, Aahan Attullah, Muneeb Ur Rehman, Kashmala Hussain, Maria Habib

**Affiliations:** 1 Surgical Oncology, Shaukat Khanum Memorial Cancer Hospital and Research Centre, Lahore, PAK; 2 General Surgery, Lady Reading Hospital (LRH) Medical Teaching Institution (MTI), Peshawar, PAK; 3 Radiology, Lady Reading Hospital (LRH) Medical Teaching Institution (MTI), Peshawar, PAK

**Keywords:** clinical outcomes, prognostic markers, disease severity, serum triglycerides, acute biliary pancreatitis

## Abstract

Background

Acute biliary pancreatitis (ABP), a major inflammatory illness, is primarily caused by gallstone blockage of the common bile duct. The pathophysiology of ABP has been linked to serum triglyceride (TG) levels, suggesting a potential role for TG in predicting disease severity.

Objective

The research objective was to investigate the association between serum TG levels and the severity of ABP.

Methodology

This retrospective cohort study sought to determine the relationship between blood TG levels and the severity of ABP. It was conducted at Lady Reading Hospital in Peshawar, Pakistan, from September 2023 to March 2024. A total of 530 ABP patients were divided into two groups based on their TG levels: normal (<150 mg/dl) and elevated (≥150 mg/dl). Clinical data were gathered, including demographics, comorbidities, laboratory results, severity ratings (APACHE II and Ranson's criteria), and clinical outcomes. Descriptive statistics, Chi-square tests, and multivariate logistic regression were used in the statistical analysis.

Results

Patients with elevated TG levels (n=130) demonstrated higher median Ranson's criteria (3.24 vs. 2.53, p<0.001) and APACHE II scores (10.53 vs. 8.73, p<0.001) compared to those with normal TG levels (n=400). Elevated TG levels were associated with increased severity of ABP, with ORs of 2.41 (95% CI: 1.23-4.74) for mild vs. severe ABP. Clinical outcomes such as ICU admission (21.54% vs. 3.25%, p<0.001), mortality (6.15% vs. 0.50%, p<0.001), and pancreatic necrosis (10.77% vs. 1.25%, p<0.001) were significantly worse in the elevated TG group.

Conclusion

Elevated serum TG levels (≥150 mg/dl) are independently associated with increased severity of ABP, as indicated by higher severity scores and poorer clinical outcomes.

## Introduction

The condition known as acute biliary pancreatitis (ABP), caused by gallstone blockage of the common bile duct, remains a major global health problem [1,2]. The complex relationship between lipid metabolism and pancreatic inflammation is illustrated by the emerging role of serum triglyceride (TG) levels in prognosticating ABP severity [3].

Pancreatitis, in general, is associated with significant risks of morbidity and mortality, and a considerable number of cases are caused by ABP [4-6]. The severity of ABP can vary greatly, from moderate, self-limiting instances to severe forms linked to systemic consequences such as organ failure and even mortality [7,8]. Identifying reliable indicators of disease severity is crucial for improving clinical management and enhancing patient outcomes [9, 10].

Due to their role in lipid metabolism and potential connection to the inflammatory mechanisms that produce ABP, serum TG levels have drawn attention [11]. Elevated TG levels may exacerbate pancreatic damage through lipotoxicity and inflammatory mediators [12]. However, further research is necessary as the exact relationship between TG levels and ABP severity is still not fully understood [13].

Serum TG levels have been investigated as predictors of ABP severity, though the results of previous research have been inconsistent [2,14]. While some studies suggest a positive correlation between higher TG levels and increased severity, others have found no significant relationship [15,16]. These discrepancies highlight the importance of conducting thorough, thoughtful research to fully understand this link.

Closing this knowledge gap is essential for several reasons. First, elucidating the role of TG levels in ABP severity may improve risk assessment techniques, aiding healthcare professionals in allocating resources and implementing early interventions. Secondly, a deeper understanding of this relationship could inform future treatment strategies aimed at mitigating the harmful effects of hypertriglyceridemia in patients with ABP.

Research objective

The research objective was to investigate the association between serum TG levels and the severity of ABP.

## Materials and methods

Study design and settings

This research utilized a retrospective cohort design and was conducted over a span of six months, from September 2023 to March 2024, at Lady Reading Hospital in Peshawar, Pakistan. As a tertiary care facility, this hospital provided the comprehensive setting required for the study, offering extensive medical services and facilities.

Inclusion and exclusion criteria

The study included patients who were at least 18 years old and hospitalized within three days of the onset of ABP. Patients were required to have their serum lipid levels, particularly TGs, checked within one day of admission. Exclusion criteria included individuals with a history of cancer, those who had previously undergone biliopancreatic surgery or experienced pancreatic trauma, patients on long-term immunosuppressants or hormones, those with terminal illnesses, pregnant or breastfeeding women, and individuals with incomplete medical records. These criteria were established to ensure a well-defined study population, thereby enhancing the accuracy and relevance of the research outcomes.

Sample size

The study included a total of 530 individuals diagnosed with ABP. Among these, 400 patients had normal blood TG levels (<150 mg/dl), while 130 patients had elevated TG levels (≥150 mg/dl).

Data collection

Clinical data were collected from electronic medical records. This included demographic information, medical history, laboratory measurements (such as serum TG levels), severity ratings (including APACHE II score and Ranson's criteria), and clinical outcomes. Skilled medical personnel ensured the completeness and accuracy of the data collected.

Severity classification

The severity of acute biliary pancreatitis was classified using the APACHE II score and Ranson's criteria. The APACHE II score categorizes severity into mild (score ≤ 8), moderate (score 9-14), and severe (score > 14). Ranson's criteria classify severity based on the presence of specific clinical and laboratory findings, with a higher number of criteria met indicating greater severity.

Statistical analysis

Statistical analysis was conducted in SPSS software version 23. Descriptive statistics were used to summarize the clinical and demographic characteristics of the study population. The chi-square test was employed to examine the association between blood TG levels and the severity of ABP concerning categorical variables. Multivariate logistic regression analysis was conducted to identify independent predictors of severe ABP, adjusting for potential confounders.

Ethical approval

Ethical approval for the study was obtained from the Institutional Review Board (IRB) of Lady Reading Hospital in Peshawar, Pakistan (LRH/MTI-IRB15738). The research adhered to the principles outlined in the Declaration of Helsinki. Given the retrospective nature of the study, informed consent was not required. Patient data were anonymized in compliance with data protection regulations to ensure patient privacy.

## Results

The study population's demographics are shown in Table 1, which contrasts patients with normal serum TG levels (n = 400) and those with high TG levels (n = 130) in cases of ABP. The age distribution of the normal TG group reveals a mean age of 46.23 ± 12.51 years: 37.75% of the group was between the ages of 18 and 40, 49.0% between 41 and 60, and 13.25% above 61. In comparison, the raised TG group had a slightly higher mean age of 48.84 ± 11.77 years, with 36.92% aged 18-40 years, 43.85% aged 41-60 years, and 19.23% aged above 61 years. In the normal TG group, there were 58.25% males and 41.75% females, whereas in the increased TG group, there were 60.00% males and 40.00% females. The distribution of genders was comparable in both groups. The normal TG group had a mean BMI of 26.51 ± 4.13 kg/m², whereas the increased TG group had a mean BMI of 27.3 ± 4.52 kg/m². Comorbidities included hypertension in 21.50% of the normal TG group and 23.85% of the elevated TG group; diabetes mellitus in 15.75% and 20.00%, respectively; chronic kidney disease in 8.00% and 13.08%; coronary artery disease in 11.75% and 16.92%; and chronic lung disease in 7.25% and 10.00%. In the normal TG group, 25.75% and in the high TG group, 33.85% reported drinking alcohol; 30.25% and 43.85% reported smoking, respectively.

**Table 1:** Demographic characteristics of study population. TG: Triglycerides.

Characteristics		Normal TG Group (n=400)	Elevated TG Group (n=130)
Age (Years) (n;%)	18-40	151 (37.75)	48 (36.92)
	41-60	196 (49.0)	57 (43.85)
	Above 61	53 (13.25)	25 (19.23)
	Mean ± SD	46.23 ± 12.51	48.84 ± 11.77
Gender (n;%)	Male	233 (58.25)	78 (60.00)
	Female	167 (41.75)	52 (40.00)
BMI (kg/m²)	Mean ± SD	26.51 ± 4.13	27.3 ± 4.52
Comorbidities (n;%)	Hypertension	86 (21.50)	31 (23.85)
	Diabetes Mellitus	63 (15.75)	26 (20.00)
	Chronic Kidney Disease	32 (8.00)	17 (13.08)
	Coronary Artery Disease	47 (11.75)	22 (16.92)
	Chronic Lung Disease	29 (7.25)	13 (10.00)
Alcohol Consumption (n;%)		103 (25.75)	44 (33.85)
Smoking (n;%)		121 (30.25)	57 (43.85)

ABP patients with normal blood TG levels (n = 400) and those with high TG levels (n = 130) are contrasted clinically and by laboratory data in Table 2. The normal TG group had median serum amylase levels of 340 U/L (IQR 250-480), whereas the high TG group had median serum amylase levels of 380 U/L (IQR 290-520) (p=0.073). The median serum lipase levels were 380 U/L (IQR 280-520) for the normal TG group and 420 U/L (IQR 310-590) for the high TG group (p=0.021). According to Ranson's criteria, the increased TG group had a mean of 3.24 ± 1.46 (p<0.001), suggesting greater severity, while the normal TG group had a mean of 2.53 ± 1.21 (p<0.001). Likewise, the raised TG group had a mean APACHE II score of 10.53 ± 3.14, which was greater than the normal TG group's 8.73 ± 2.37 (p<0.001). The normal TG group had serum total cholesterol levels of 180.67 ± 40.39 mg/dl, whereas the high TG group had values of 200.36 ± 45.41 mg/dl (p=0.012). Increased total cholesterol (TG) was linked to decreased HDL cholesterol (45.97 ± 12.75 mg/dl vs. 50.34 ± 10.72 mg/dl, p=0.027) and increased serum LDL cholesterol (130.74 ± 35.13 mg/dl vs. 110.59 ± 30.88 mg/dl, p=0.008). The raised TG group had a longer hospital stay (8.32 ± 3.61 days) than the normal TG group (6.13 ± 2.44 days, p<0.001).

**Table 2:** Clinical characteristics and laboratory parameters. U/L: Units per liter; mg/dl: Milligrams per deciliter; TG: Triglycerides; APACHE II: Acute Physiology and Chronic Health Evaluation II; LDL: Low-density lipoprotein; HDL: High-density lipoprotein.

Parameter	Normal TG Group (n=400)	Elevated TG Group (n=130)	P-value
Serum Amylase (U/L), median (IQR)	340 (250-480)	380 (290-520)	0.073
Serum Lipase (U/L), median (IQR)	380 (280-520)	420 (310-590)	0.021
Ranson's Criteria, Mean ± SD	2.53 ± 1.21	3.24 ± 1.46	<0.001
APACHE II Score, Mean ± SD	8.73 ± 2.37	10.53 ± 3.14	<0.001
Serum Total Cholesterol (mg/dl), Mean ± SD	180.67 ± 40.39	200.36 ± 45.41	0.012
Serum LDL Cholesterol (mg/dl), Mean ± SD	110.59 ± 30.88	130.74 ± 35.13	0.008
Serum HDL Cholesterol (mg/dl), Mean ± SD	50.34 ± 10.72	45.97 ± 12.75	0.027
Length of Hospital Stay (days), Mean ± SD	6.13 ± 2.44	8.32 ± 3.61	<0.001

**Figure 1:**
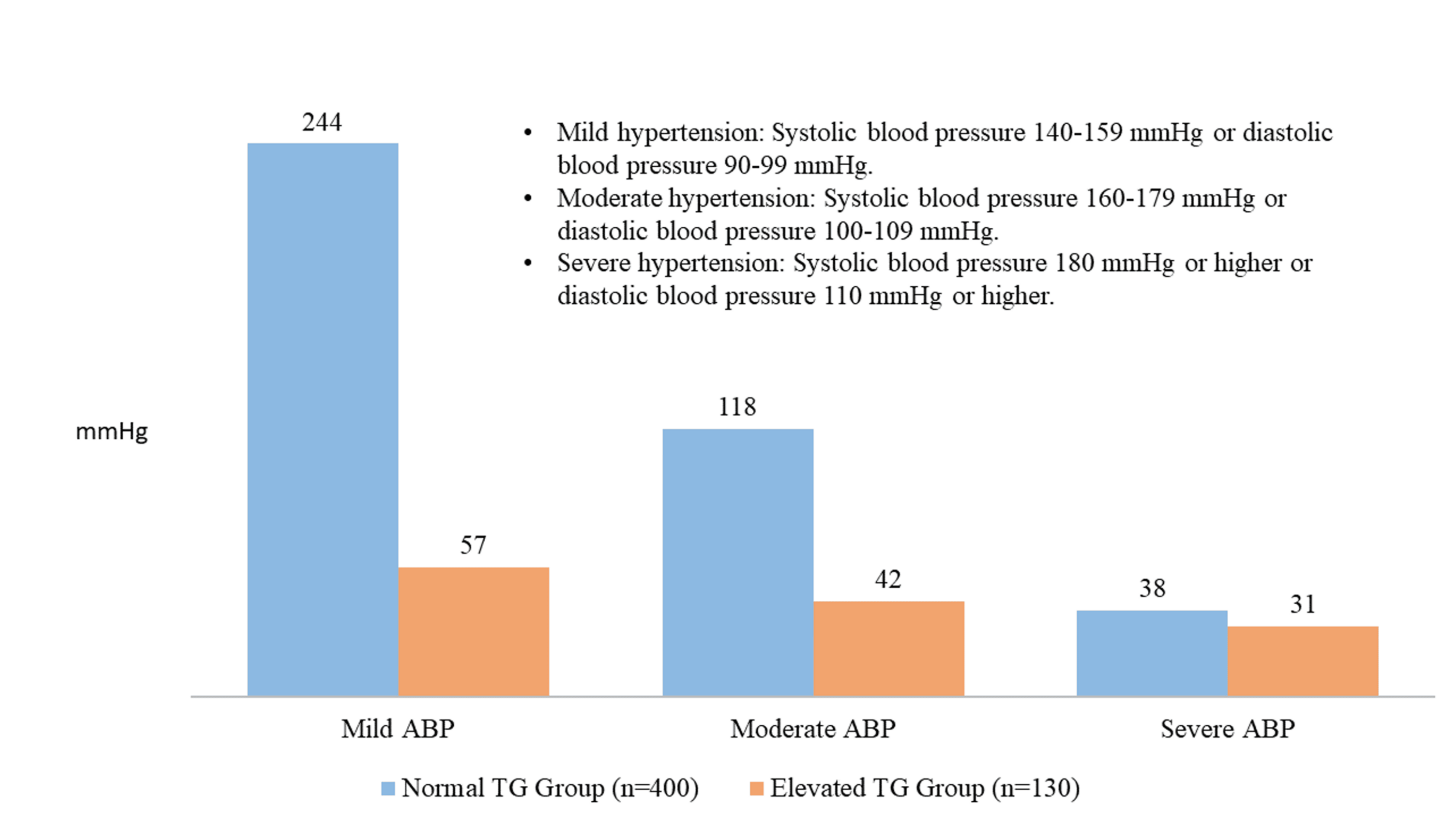
Distribution of ABP severity by serum TG levels. TG: Triglyceride; ABP: Acute biliary pancreatitis.

The distribution of serum TG levels-stratified ABP severity levels is shown in Figure 1. Of the 400 patients in the Normal TG Group, 61.00% (n = 244) had mild ABP, 29.50% (n = 118) had moderate ABP, and 9.50% (n = 38) had severe ABP. Conversely, 43.85% (n=57) of patients in the Elevated TG Group (n=130) had mild ABP, 32.31% (n=42) had moderate ABP, and 23.85% (n=31) had severe ABP.

The relationship between the severity of ABP and serum TG levels is shown in Table 3 of a cohort study conducted at Lady Reading Hospital. The odds ratio (OR) in the normal TG group (<150 mg/dl) was 0.52 (95% CI 0.30-0.90) for mild vs severe ABP and 0.75 (95% CI 0.51-1.11) for mild vs moderate ABP. Conversely, the OR for mild vs moderate ABP was 1.20 (95% CI 0.71-2.03) and for mild vs severe ABP was 2.41 (95% CI 1.23-4.74) in the increased TG group (≥150 mg/dl).

**Table 3:** Association Between Serum TG Levels and ABP Severity

Severity Level vs. TG Level	Normal TG Group (n=400)	Elevated TG Group (n=130)	p-value
Mild vs. Moderate ABP	OR 0.75 (95% CI 0.51-1.11)	OR 1.20 (95% CI 0.71-2.03)	0.312
Mild vs. Severe ABP	OR 0.52 (95% CI 0.30-0.90)	OR 2.41 (95% CI 1.23-4.74)	0.012
Moderate vs. Severe ABP	OR 0.69 (95% CI 0.35-1.35)	OR 2.00 (95% CI 1.01-3.96)	0.047

According to these results, compared to normal TG levels, higher TG levels are strongly linked to an increased risk of severe ABP (p=0.012). This link was further supported by adjusted multivariate logistic regression analysis (Table 4), which revealed an odds ratio (OR) of 2.18 (95% CI 1.17-4.06) for severe ABP in the increased TG group. The findings from a multivariate logistic regression analysis evaluating risk factors for severe ABP are shown in Table 4. Elevated serum TG levels (≥150 mg/dl) indicated a significant association with severe ABP, with an OR of 2.18 (95% CI 1.17-4.06, p=0.015), suggesting a more than twofold increase in the likelihood of severe ABP compared to lower TG levels. With an OR of 1.43 (95% CI 1.25-1.64, p<0.001), the APACHE II score also demonstrated a strong correlation, indicating that higher scores are predictive of greater severity. Age showed a weak correlation (OR of 1.02, 95% CI 0.98-1.06, p = 0.287). Furthermore, a significant correlation was found between elevated blood lipase levels and severe ABP, with an OR of 1.08 (95% CI 1.02-1.14, p=0.007). Interestingly, there appears to be a protective effect as higher blood total cholesterol levels were inversely associated with severe ABP, with an OR of 0.98 (95% CI 0.97-0.99, p=0.002).

**Table 4:** Multivariate logistic regression analysis of factors associated with severe ABP. TG: Triglycerides; APACHE II: Acute Physiology and Chronic Health Evaluation II; ABP: Acute Biliary Pancreatitis.

Variable	OR (95% CI)	P-value
Elevated TG (≥150 mg/dl)	2.18 (1.17-4.06)	0.015
APACHE II Score	1.43 (1.25-1.64)	<0.001
Age (years)	1.02 (0.98-1.06)	0.287
Serum Lipase Levels	1.08 (1.02-1.14)	0.007
Serum Total Cholesterol	0.98 (0.97-0.99)	0.002

The clinical outcomes for patients with ABP are summarized in Table 5, based on serum TG levels. There was a significant difference (p<0.001) in the number of patients requiring admission to the intensive care unit (ICU) between the normal TG group (<150 mg/dl) and the raised TG group (≥150 mg/dl), with 13 patients (3.25%) in the former group and 28 patients (21.54%) in the latter. Additionally, the raised TG group had significantly higher mortality rates-8 deaths (6.15%) compared to 2 deaths (0.50%) in the normal TG group (p<0.001). Comparatively, five patients (1.25%) in the normal TG group and 14 patients (10.77%) in the increased TG group experienced pancreatic necrosis (p<0.001).

**Table 5:** Clinical outcomes based on serum TG levels. TG: Triglycerides.

Outcome	Normal TG Group (n=400)	Elevated TG Group (n=130)	P-value
ICU Admission (n;%)	13 (3.25%)	28 (21.54%)	<0.001
Mortality (n;%)	2 (0.50%)	8 (6.15%)	<0.001
Pancreatic Necrosis (n;%)	5 (1.25%)	14 (10.77%)	<0.001

**Figure 2:**
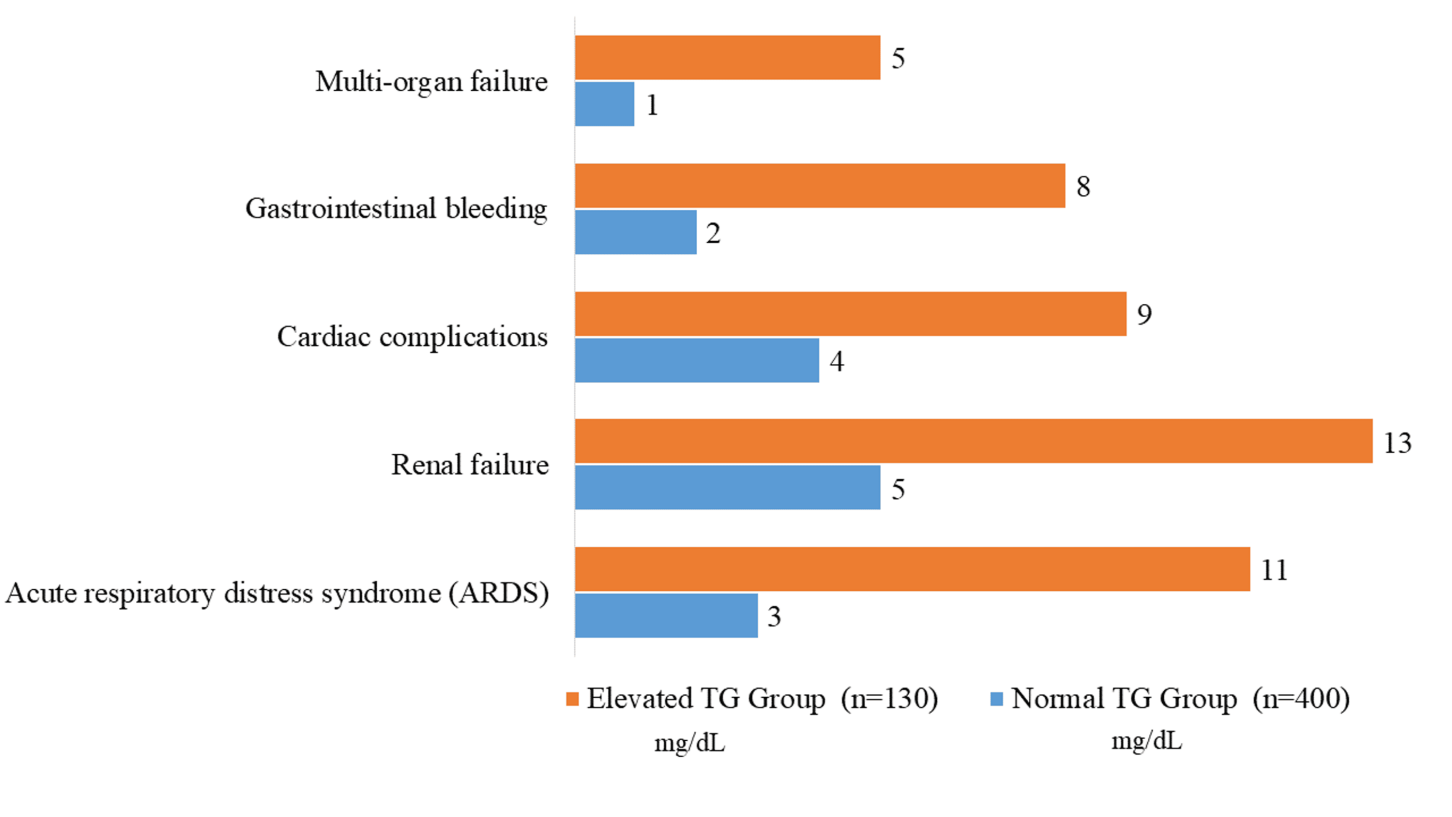
Complications during hospital stay. TG: Triglycerides.

According to blood TG levels, Figure 2 displays the frequency of complications during hospital stays for patients diagnosed with ABP. Within the normal TG group (<150 mg/dl), three patients experienced acute respiratory distress syndrome (ARDS), five underwent renal failure, four had cardiac complications, two suffered gastrointestinal hemorrhage, and one had multi-organ failure. On the other hand, these events were more common in the increased TG group (≥150 mg/dl), with 11 instances of ARDS, 13 cases of renal failure, nine cases of cardiac complications, eight cases of gastrointestinal hemorrhage, and five cases of multi-organ failure. These results highlight the increased risk of serious complications in ABP patients with elevated blood TG levels, pointing to a potential link between hypertriglyceridemia and worse clinical outcomes in this patient group.

## Discussion

The association between serum TG levels and the severity of ABP has been a subject of ongoing research due to its potential implications for clinical management and patient outcomes. The results of this research showed that raised TG levels (≥150 mg/dl) were substantially linked to greater ABP severity, as shown by higher median APACHE II and Ranson's criterion scores in the elevated TG group than in the normal TG group. To be more precise, patients with higher TG levels had mean Ranson's criterion scores of 3.24 ± 1.46 and APACHE II scores of 10.53 ± 3.14, whereas individuals with normal TG levels scored lower (Ranson's criteria: 2.53 ± 1.21; APACHE II score: 8.73 ± 2.37). This is consistent with other studies showing a relationship between elevated TG levels and both the severity of pancreatitis episodes and pancreatic inflammation [14, 17].

Additionally, the investigation revealed variations in lipid profiles between TG groups, suggesting plausible processes behind the correlation. Compared to patients with normal TG levels (LDL: 110.59 ± 30.88 mg/dl; HDL: 50.34 ± 10.72 mg/dl), patients with increased TG levels had lower HDL cholesterol (45.97 ± 12.75 mg/dl) and higher serum LDL cholesterol (130.74 ± 35.13 mg/dl). Increased oxidative stress and inflammatory responses have been connected to these lipid changes, which may exacerbate pancreatic damage in ABP [18, 19].

Apart from biochemical indicators, clinical outcomes also highlighted the influence of higher TG levels on the severity of ABP. Compared to the normal TG group (6.13 ± 2.44 days), the increased TG group had a substantially longer mean hospital stay (8.32 ± 3.61 days). This extended hospital stay is consistent with other research indicating that hypertriglyceridemia may be a factor in the slower rate of recovery and higher use of medical resources in cases with acute pancreatitis [20, 21].

The results of the research also showed that fatality rates and the need for critical care varied significantly according to TG levels. Compared to individuals with normal TG levels (3.25%), a higher percentage of patients with raised TG levels (21.54%) required admission to the intensive care unit (ICU), and their corresponding death rates were 6.15% and 0.50%, respectively. These results, which support related findings in the literature, emphasize the clinical relevance of TG levels as a potential predictor of serious complications and a poor prognosis in ABP [22, 23].

Moreover, high TG levels (≥150 mg/dl) were shown to be an independent predictor of severe ABP (OR 2.18, 95% CI 1.17-4.06, p=0.015), according to the study’s multivariate logistic regression analysis. This bolsters the body of research suggesting that TG levels may be an important prognostic indicator for risk assessment and timely intervention in the treatment of ABP [24].

However, several limitations should be acknowledged in interpreting the findings of this study. Firstly, the research was conducted at a single center, which may limit the generalizability of the results to broader populations with ABP. Secondly, the study primarily relied on retrospective data collection, which could introduce biases and incomplete information. Additionally, despite controlling for various confounding factors, the observational nature of the study cannot establish causality between elevated TG levels and ABP severity. Future prospective studies involving diverse patient cohorts and employing standardized protocols are warranted to validate these associations and elucidate underlying mechanisms more comprehensively.

## Conclusions

This research underscores the strong correlation between high blood TG levels and the severity of ABP. The results indicate that patients with TG levels of 150 mg/dl or greater exhibit more severe clinical symptoms, evidenced by elevated Ranson's criteria and APACHE II scores, longer hospital stays, higher ICU admission rates, and increased mortality compared to those with normal TG levels. These findings illuminate how lipid metabolism exacerbates pancreatic inflammation and highlight the potential utility of TG levels as a predictive marker to guide early intervention strategies for improved outcomes in individuals with acute pancreatitis.
